# Formation of Self-Generated Gradients of Iodixanol

**DOI:** 10.1100/tsw.2002.284

**Published:** 2002-05-16

**Authors:** John Graham

**Affiliations:** School of Biomolecular Sciences, Liverpool John Moores University, UK

**Keywords:** self-generated gradient, OptiPrep, iodixanol, vertical rotor, near-vertical rotor, fixed-angle rotor, centrifugation time, RCF, temperature

## Abstract

The formation of self-generated gradients of iodixanol from a solution of uniform concentration requires the use of vertical or near-vertical rotors. The density profile that is generated depends upon the sedimentation path length of the rotor, centrifugation time, RCF and temperature. Modulation of the starting concentration changes the density range of the gradient. This Protocol Article illustrates the effect of these parameters on gradient shape in a few selected rotors. Because the gradients are formed by the centrifugal field, they are highly reproducible and easy to execute.

**METHOD TYPE:** extraction, isolation, purification and separation

**SUB METHOD TYPE:** centrifugation

Self-generated gradients provide a simple and rapid means of fractionating membrane vesicles, some organelles, macromolecules, and macromolecular complexes. They facilitate the processing of multiple samples and the density profiles generated are highly reproducible under defined centrifugation conditions.

Although vertical or near-vertical (8–9$^{o}$ tube slope) are the preferred rotors, low-angle fixed-angle rotors can be used. Swinging-bucket rotors are never used for self-generated gradients.

The shape of the gradient that is formed depends on a number of factors: (1) the sedimentation path length of the rotor, (2) the RCF, (3) the time of centrifugation, (4) the temperature, and (5) the starting concentration of iodixanol.

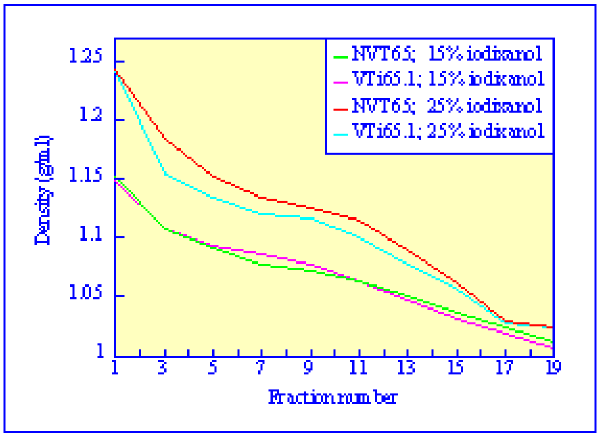

**FIGURE 1**. Density profiles: 350,000 *g* at 4°C for 3 h.

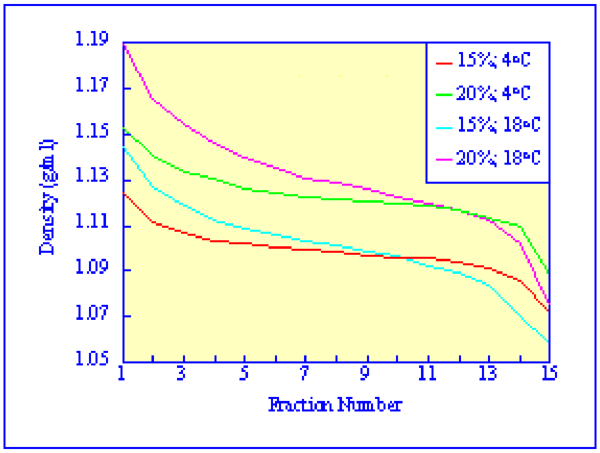

**FIGURE 2**. Density profiles: 350,000 *g* for 1 h in NVT65.

Fig. 1 compares the gradient profiles from two Beckman rotors, which provide the optimum conditions for gradient formation, the VTi65.1 (vertical) and NVT65 (near-vertical); their path lengths are approx. 17 and 25 mm, respectively. The shape of gradient profiles obtained at the two iodixanol starting concentrations is similar. After centrifugation at 350,000 $g_{av}$ at 4°C for 3 h, the gradients are approximately linear over most of the density range.

Fig. 2 shows that even after as short a time as 1 h, a useful gradient can be obtained in the NVT65 at 350,000 $g_{av}$, at 18°C. As the centrifugation time is reduced, so the gradient becomes more sigmoidal, steep at both ends and more shallow in the middle. Reducing the temperature to 4°C has a significant effect on the density profile at these short times. Lowering the temperature reduces the rate of sedimentation of solute molecules, so gradients are more linear at higher temperatures.

In all cases the density range of the gradient is controlled by the starting concentration of the iodixanol, but this factor has relatively little influence on the shape of the gradient. The density range will also be affected by the density and viscosity of the osmotic balancer that is used to dilute the OptiPrep™.

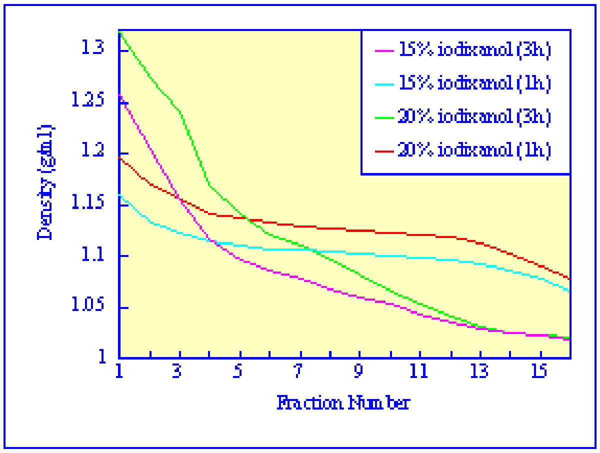

**FIGURE 3**. Density profiles: 350,000 *g* at 15°C in TLN 100.

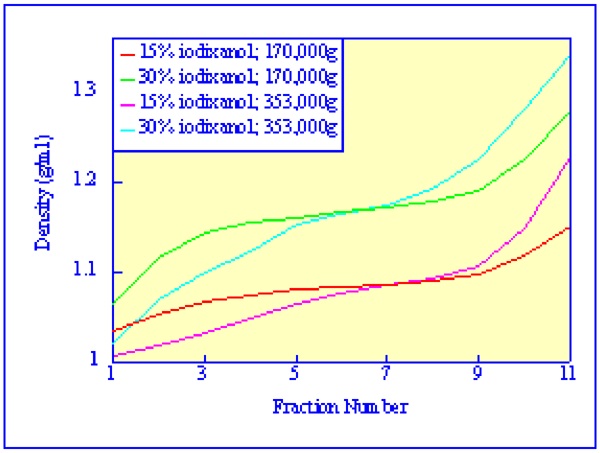

**FIGURE 4**. Density profiles: VTi65.1 rotor centrifuged for 3 h at 15°C.

Time of centrifugation has a significant effect on density profile. Fig. 3 shows the typical effect of increasing the time of centrifugation in the TLN 100 rotor. The density profile changes from sigmoidal to one which is more-or-less linear over the top two thirds of the gradient and then becomes steeper in the bottom third. Although the TLN 100 holds much smaller volume tubes (3–4 ml) than either the VTi65.1 or NVT65 (11–13 ml), its sedimentation path length is rather similar and hence its gradient generating capacity is rather similar.

S-shaped gradients are also formed at lower RCFs (see Fig. 4) and the effect of RCF on the density profile is similar at both low and high concentrations of iodixanol. At each RCF there is an equilibrium situation when the rate of sedimentation of iodixanol is exactly balanced by its back diffusion so that increasing the centrifugation time has relatively little effect on the profile. For the VTi65.1 rotor this time is approx. 4 h.

Some fixed-angle rotors can be used for self-generated iodixanol gradients, particularly the smaller volume low angle rotors. Fig. 5 shows that at 180,000 g and above, the Kontron 70.38 fixed-angle rotor has an unusually low angle (20°) and a tube volume of approx. 11 ml. Other fixed-angle rotors, even those capable of higher RCFs are often less suited because generally their higher angles means that they have longer sedimentation path lengths. Some Beckman fixed-angle rotors can be adapted to smaller volumes using g-Max adapters, which also effectively reduce the path length.

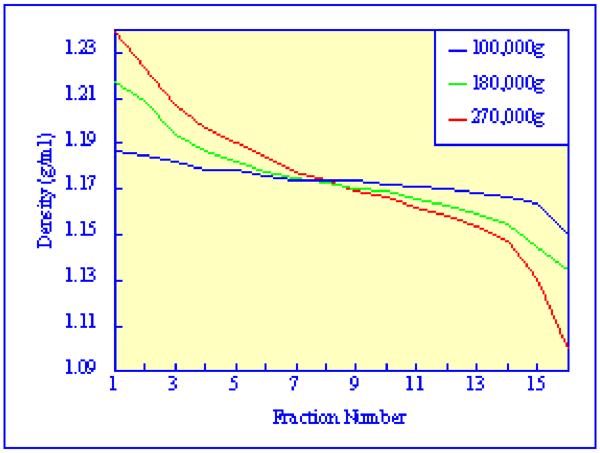

**FIGURE 5**. Density profiles: 30% iodixanol in a Kontron 70.38 rotor, centrifuged for 3 h at 4°C at 180,000 *g*.

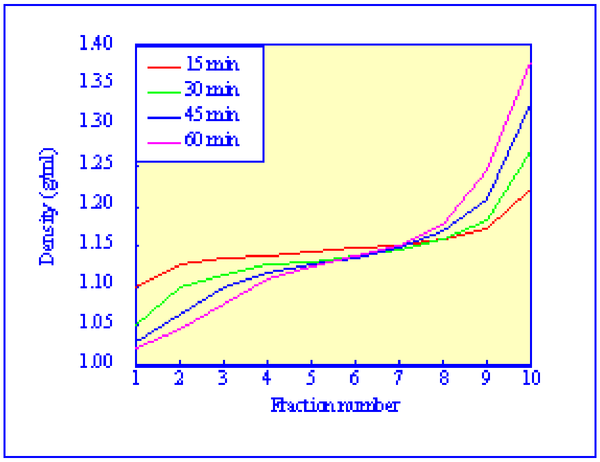

**FIGURE 6**. Density profiles: Sorvall S150-AT fixed-angle rotor from 25% iodixanol at 710,000 *g* at 16°C.

Small volume fixed-angle rotors for microultracentrifuges can be used very effectively for self-generated gradients, particularly with the very high RCFs, which can now be achieved with these machines. It is possible to create self-generated gradients in less than 1 h at over 700,000 g (see Fig. 6).

In this Protocol Article all iodixanol concentrations are given as % (w/v) and all RCF values as $g_{av}$.

## ACKNOWLEDGEMENTS

The author and TheScientificWorld wish to thank Axis-Shield PoC, AS, Oslo, Norway for their kind permission to adapt OptiPrep™ Application Sheet S4 in the preparation of this Protocol Article.
